# A botanical extract blend of *Mangifera indica* and *Sphaeranthus indicus* combined with resistance exercise training improves muscle strength and endurance over exercise alone in young men: a randomized, blinded, placebo-controlled trial

**DOI:** 10.3389/fnut.2024.1393917

**Published:** 2024-05-03

**Authors:** Dawna Salter, Shubhatara Swamy, Kevin Manohar Salis, Dheeraj Kumar Deep, Pratibha Nadig

**Affiliations:** ^1^Department of Clinical Research and Innovation, PLT Health Solutions, Inc., Morristown, NJ, United States; ^2^Department of Pharmacology, Vydehi Institute of Medical Sciences and Research Centre, Bengaluru, India; ^3^D2L Clinical Solutions, Bengaluru, India

**Keywords:** 1-RM, mTOR, sports performance, testosterone, dietary supplement

## Abstract

Resistance exercise training (RET) is used to improve muscular strength and function. This study tested the hypothesis that RET alongside daily supplementation of a *Sphaeranthus indicus* and *Mangifera indica* extract blend (SMI) would augment bench press (BP) and leg extension (LE) strength and repetitions to failure (RTF) compared to RET alone. Ninety-nine men (age 22 ± 3) completed the trial after randomization into one of four groups: (A1) 425 mg SMI plus one RET set; (A2) 850 mg SMI plus one RET set; (P1) placebo plus one RET set; and (P2) placebo plus two RET sets. RET sets were 6–8 BP and LE repetitions at 80% of a progressive one repetition maximum (1-RM), performed 3x/week for 8 weeks. Strength and RTF were evaluated at baseline and days 14, 28, and 56 while serum values of total testosterone (TT), free testosterone (FT), and cortisol (C) values were evaluated at baseline and day 56. RET significantly (*p* < 0.05) increased 1-RM, RTF, and T measures above baselines regardless of group assignment, but the increases were greater in the supplemented groups. At week 8, A1 bench pressed more than P1 (71.5.5 ± 17.5 kg vs. 62.0 ± 15.3 kg, *p* = 0.003), while A2 pressed 13.8 ± 3.0 kg more (95% CI 5.7–21.8, *p* < 0.001) than P1 and 9.9 ± 13.0 kg more (95% CI 1.7–18.2, *p* = 0.01) than P2. Also at week 8, the mean LE 1-RM of A1 (159.4 ± 22.6 kg) and A2 (162.2 ± 22.9 kg) was greater (*p* < 0.05) than that of P1 (142.2 ± 25.6 kg) and P2 (146.5 ± 19.7 kg). Supplementation improved RTF, TT, and FT values over those measured in exercise alone (*p* < 0.05), while C levels in A2 (9.3 ± 3.8 μg/dL) were lower than P2 (11.7 ± 3.8 μg/dL, *p* < 0.05). Daily supplementation with SMI was well tolerated and may help optimize muscle adaptive responses to RET in men.

## Introduction

Progressive resistance exercise training (RET) is a well-established exercise method used to boost muscle strength and improve athletic outcomes (1–3) but it also improves muscularity and appearance, decreases disability, and supports better overall health for non-athletes (4–6). Muscle fibers contract against weighted loads during RET, creating mechanical signals that converge alongside other intracellular signals onto the cellular protein kinase mTOR (the mechanistic target of rapamycin) (7), a metabolic regulator intimately involved in directing the rate of muscle protein synthesis post-exercise (8, 9). However, RET-generated mechanical signaling can result in dramatically different strength outcomes between individuals despite their training being of similar intensity (10, 11), so those engaged in weight lifting often manipulate training variables, modify their diet, and/or use dietary supplements in an attempt to facilitate anabolic responses to their training efforts. High-protein diets, protein supplements (12–14), and supplements such as beta-hydroxy beta-methyl butyrate (HMB) and creatine (15–17) can help optimize post-exercise muscle protein synthesis. Additionally, safe, conveniently dosed, plant-based products are increasingly being explored for their ability to support RET responses.

Aiming to develop a scientifically supported natural product to impact muscle function, a series of botanical extracts were screened for their ability to modify key mechanisms involved in muscle metabolism. Two extracts showing efficacies across the screening assays were those derived from *Sphaeranthus indicus* (East Indian Globe Thistle) flower head and *Mangifera indica* (Mango Tree) bark, both preparations that have been widely used in Indian Ayurvedic medicine to target immune modulation, analgesia, antioxidant, anxiolytic, anti-inflammatory, and other activities (18, 19). When tested in a variety of unpublished *in vitro* experiments, a specific 2:1 preparation of *S. indicus* flower head and *M. indica* bark extracts consistently activated mTOR and upregulated muscle-specific transcription factors, including myogenin and myoD, in skeletal muscle cells, improved measures of mitochondrial function in myoblasts, and synergistically enhanced nitric oxide (NO) generation in endothelial cells. After preclinical validation for dosing and safety (20), a 650 mg dose of this botanical blend was clinically tested in a cohort of recreationally active young men participating in 8 weeks of a training regimen incorporating chest/shoulder, back, leg, and arm exercises. These subjects improved measures of upper and lower body strength compared to an exercising group given a placebo, suggesting this extract blend may improve the processes underlying muscle adaptive responses to RET (21). It is unknown if a comparable (850 mg) or half-dose (425 mg) of a water-soluble version of this same 2:1 extract combination of *S. indicus* L. flower head and *M. indica* bark (SMI) may also modulate those signaling pathways that direct and support muscle protein synthesis post-RET. In the present study, two doses (850 and 425 mg) of SMI are investigated for their ability to augment muscle strength and endurance in RET-naïve, healthy young men undergoing eight weeks of supervised RET compared to two exercising placebo groups, one completing an exercise protocol matching that of the supplemented groups, and one completing double the number of RET sets. Serum values of Free Testosterone (FT), total testosterone (TT), and cortisol (C) were also evaluated in this study.

## Materials and methods

### Ethics approval and registration

This randomized, double-blind, placebo-controlled study was conducted at an independent research organization according to the Declaration of Helsinki in agreement with the International Conference on Harmonization guidelines on Good Clinical Practice. The study protocol was approved by the Institutional Ethics Committee (IEC) of the Vydehi Institute of Medical Sciences and Research Center (Bengaluru, Karnataka, India) and registered in the Clinical Trials Registry (CTRI) of India (Registration no. CTRI/2018/12/016641).

### Study participants

Healthy men, 19–29 years, with a body mass index (BMI) of 18.5–29.9 kg/m^2^, with a normal electrocardiogram, and who were previously untrained in resistance or power exercise for at least 6 months, were recruited for the study. Men with a history of alcohol or drug abuse, sleep disorder, eating disorder, or psychiatric condition, or those needing to fast as a personal practice, were excluded from the study. Additional exclusions included men who used protein or had taken any dietary supplements containing protein, creatine, HMB, or other supplements thought to improve muscle strength and/or muscle mass, or used corticosteroids, testosterone, or anabolic drugs in the three months before the study initiation. Each volunteer was aware of the study protocol and provided written informed consent before any study-related procedures were conducted.

### Study design

A total of 120 subjects were enrolled and randomized into one of four equal groups (*n* = 30) by following the randomization codes as generated by SAS procedure PROC PLAN using block design: (A1) 425 mg SMI plus one RET set; (A2) 850 mg SMI plus one RET set; (P1) placebo plus one RET set; and (P2) placebo plus two RET sets. This study involved five scheduled visits, screening (days −14 to −1), randomization/baseline (day 1), and days 14, 28, and 56. The study products were labeled by randomization code and dispensed to the participants on study days 1, 14, and 28. Subjects were instructed to consume two capsules every morning for 56 days. Compliance with the supplementation regimen was monitored through daily diary recordings of capsule consumption and the collection/counting of all unused capsules. All subjects were provided a training regimen as per their group assignment and compliance with the training regimen was calculated as the total number of sessions completed under the supervision of trained instructors and study personnel per week for 8 weeks. Subjects were instructed to maintain their habitual dietary intake and physical activity levels, except for their assigned RET program, during the study. Participants completed written 3-day diet records for two weekdays and one weekend day at pretraining and in the last week of the training period and these diet records were evaluated for consistent eating patterns. Adverse events, tolerance, and vital signs—heart rate, blood pressure, temperature, respiratory rate—were assessed throughout the study and recorded at each study visit. Body weights were measured with a standard scale in triplicate to the nearest 0.1 kg and the average was recorded at each visit. Fasting blood samples were collected at screening, baseline, and day 56. Performance testing occurred at baseline and on days 14, 28, and 56. All participants, investigators, and study personnel remained blinded to treatment assignment for the duration of the study.

### Supplementation

The botanical supplement, SMI, is a water-dispersible version of a formulation containing a blend of extracts from *S. indicus* flower heads and *M. indica* L. stem bark at 50.03% (w/w) herbal blend and 49.97% (w/w) excipients. The excipients include dewaxed shellac (33.3%), guar gum (8.33%), citric acid (6.67%), and colloidal silicon dioxide (1.67%). The final product was standardized to contain not less than 2.5% 7-hydroxy frullanolide and 2% mangiferin following methods of preparation and analytical procedures as previously described for the non-water dispersible formulation (20). The investigational products and placebo were manufactured under a strict Good Manufacturing Process, packaged, and labeled by randomization code by Laila Nutraceuticals (Vijayawada, India). SMI is commercially available as Myotor^®^ and RipFACTOR™ from PLT Health Solutions (Morristown, NJ, USA).

### Exercise training program

The two active groups (A1, A2) and one placebo group (P1) completed the same exercise protocol, whereas the second placebo group (P2) completed double the number of RET sets. A second placebo group completing double the number of RET sets was deployed as a type of positive control, as research supports that an increased volume of weighted-load training can increase strength gains in a dose-response relationship (1, 3). Given the potential for uncaptured differences in individual work efforts within our cohort of untrained subjects, we leveraged this four-group experimental design with two exercise-dose comparator groups to help ensure any measured strength differences between treatment and placebo could be attributed to treatment effects.

All the volunteers completed RET on three non-consecutive days per week for 8 weeks under the supervision of a certified trainer and spotter. At each of the 24 training sessions, the subjects appropriately warmed up and then completed one set (for groups A1, A2, and P1) or two sets (group P2) of 6–8 repetitions of both bench press (BP) and leg extension (LE), at 80% of their most recent individualized muscle strength score (1-RM) assessment. The 1-RM, determined for each subject as the heaviest lift completed within a series of single repetitions of progressively heavier weights until failure, was assessed at baseline, day 14, and day 28. The number of repetitions and sets remained constant at each training session as per the assigned group.

### Outcome measures

The primary endpoint was the change in muscle strength, as measured by 1-RM in BP and LE exercises, from baseline to the end of the study. Each subject’s 1-RM for BP and LE was summed to obtain a measure of total muscle strength. Secondary endpoints were changes in muscle endurance, measured as the number of repetitions lifted at 80% of the subject’s baseline 1-RM until failure, and changes in serum concentrations of FT, TT, and C from baseline to day 56.

Safety parameters, including complete blood cell counts and blood chemistry— liver function tests, renal function tests, plasma total cholesterol, low- and high-density lipoprotein (LDL, HDL) cholesterol, triglycerides, and fasting glucose—were assessed from fasting blood samples taken at screening and day 56. Test parameters for urine analysis included specific gravity, pH, albumin, bile salts, bile pigment, glucose, red blood cells, and ketones.

### Muscle strength (1-RM) assessment

Muscle strength was determined by 1-RM measurements for BP (Olympic Flat Bench) and LE (StayFit Single Station) on days 0, 14, 28, and 56. Strength assessments followed the American Society of Exercise Physiologists’ procedure for accurate assessment of muscular strength (22) and were done under the supervision of a certified athletic trainer and spotters. Standardized instructions were provided on lifting techniques and testing procedures, standardized weights and bars were utilized, and verbal encouragement was provided during testing. After an appropriate whole-body warm-up, subjects performed two practice sets of 6–8 repetitions at approximately 60 and 80% of their estimated 1-RM. Subsequent lifts were single repetitions of progressively heavier weights until failure. The 1-RM was determined after three to five attempts at the heaviest single repetition weight, with a rest interval of 2–4 min between attempts.

### Muscle endurance (repetitions to failure) assessment

Muscle endurance was measured as repetitions to failure (RTF). RTF was determined by measuring the maximal number of repetitions a subject completed when lifting 80% of the established baseline 1-RM until failure. To assess a fixed measure of relative muscular endurance, the original baseline 1-RM was always utilized for muscular endurance testing throughout the study.

### Serum hormone assessment

Serum hormones were measured from fasting blood samples collected by direct vein puncture using standard enzyme immunoassay (ELISA) kits. The assay procedures for measuring TT, FT, (DRG International, Inc. Springfield, NJ; TT intra-assay coefficient of variation [CV] 3.3–4.2%, inter-assay CV 4.7–9.9%, sensitivity 0.083 ng/ml; FT intra-assay CV < 10%, inter-assay CV < 10%, sensitivity 0.06 pg/mL), and C (Cal Biotech Inc., El Cajon, CA; sensitivity 20 ng/mL) followed the protocols provided by the vendor. Each serum sample was tested in duplicate. The ELISAs were based on the principles of sandwich immuno-enzymatic reactions. The supplied substrate solutions developed the color reactions, and a microplate reader (Bio-Rad Laboratories, Hercules, CA) recorded the absorbance. The analyte concentrations in the serum samples were calculated from the standard curves plotted in each assay. The assay variation was calculated across 4 plates of 5 (FT) or 6 (TT and C) standards, and the intra- and inter-assay CVs were 0.7 ± 0.4% and 4.7 ± 0.5% for FT; 1.3 ± 0.6% and 3.8 ± 0.4% for TT; and 1.5 ± 0.9% and 10.7 ± 1.9% for C.

### Clinical laboratory and safety parameter assessment

Urine, serum, and whole blood parameters were assessed in all participants at the baseline and final visits. Biochemical parameters were measured using Cobas C 311 (Roche Diagnostics, Rotkreuz, Switzerland), and hematological parameters were measured using the auto hematology analyzer Mindray BC-20 (Shenzhen Mindray Bio-Medical Electronics Co., Ltd, China). Urine analysis was carried out using standard Siemens Multistix 10 SG reagent strips and by microscopy of sediment.

### Statistical analysis

The data were analyzed using SPSS Software version 29.0 (SPSS, Inc., Chicago, IL, USA). The normality of the data was assessed using the Shapiro-Wilk test. Where assumptions were met, data were analyzed by a 4 × 3 mixed factorial group (A1, A2, P1, P2) by time (14, 28, 56 days) ANCOVA or a univariate ANOVA (day 56) with the baseline measurement held as a covariate. Main effects (time or group) and interaction (time × group) effects were evaluated after the degrees of freedom were corrected by Greenhouse-Geisser for sphericity. The calculation of partial effect size (η^2^) was used to clarify the magnitude of the main effects, defined as small, 0.01; medium, 0.06; and large, 0.14. When the F-ratio was significant for main or interaction effects, *a priori* planned pairwise comparisons at each time point were conducted using paired (intragroup) or unpaired (intergroup) Student *t*-tests adjusted for multiple comparisons using the Bonferroni correction. Data not meeting normality assumptions were analyzed with non-parametric methods: Friedman repeated measure ANOVA by rank for within-group effects and Kruskal–Wallis ANOVA to compare groups at the same time point, adjusted by Bonferroni correction for multiple tests. Effects were considered significant at *p* < 0.05. General characteristics and descriptive statistics are expressed as mean ± standard deviation (SD), whereas the estimated marginal means and calculated mean differences (MD) are presented as means ± standard error of the measurement (SE) and reported in conjunction with the 95% confidence interval (CI).

**FIGURE 1:**
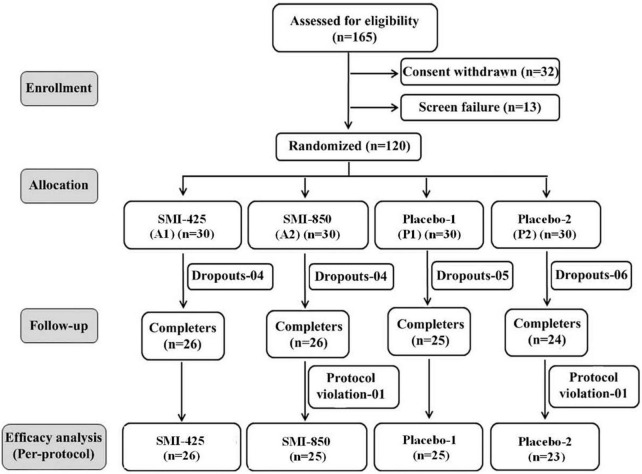
Consort diagram of participant flow through each stage of study.

*A priori* analysis utilizing pilot data determined that a sample size of at least 18 subjects per group would be required to achieve 80% power to detect a significant difference at 0.05 alpha level. With an estimated dropout rate of 20–30%, recruitment was determined at 120 subjects. One hundred and sixty-five participants were assessed for eligibility, 13 failed screening, and 32 voluntarily withdrew. After 120 subjects were randomized into 4 equal groups (*n* = 30), eighteen subjects dropped out due to scheduling difficulties before supplementation began, one dropped out during the training phase because of a non-study-related adverse event, and two subjects were removed due to protocol deviations. In total, 99 subjects completed the study: A1, *n* = 26; A2, *n* = 25; P1, *n* = 25; and P2; *n* = 23. The flow of the study progress is shown in the consort diagram (Figure 1).

## Results

### Participant characteristics, adverse events, safety, and compliance

The four groups were comparable in age, anthropometric data, and baseline measures of muscle strength, endurance, and hormones before the treatment began (Table 1). There were no serious adverse events reported during the study. Eight subjects experienced minor adverse events which were evenly divided between placebo and supplement. Specific minor adverse events in the supplemented groups included two subjects with superficial limb pain/abrasions due to minor incidents occurring outside the study, and two with generalized body aches, headache, fever, and abdominal discomfort. Adverse events within the placebo groups consisted of one subject with minor symptoms resulting from a scorpion sting, one with complaints of itching, and two with generalized body pain, fever, headache, and chills. These adverse events were self-limiting and resolved fully during the study. All subjects completed 100% of their assigned training sessions and daily consumption of study products was > 90%. Written diet records indicated dietary patterns did not alter from the beginning to the end of the study, and body weights were stable or slightly increased (Table 2). Safety parameters and vital signs were within normal clinical ranges at baseline and the end of the study (Supplementary Tables 1, 2).

**TABLE 1:** Participant characteristics at baseline.

Parameters	A1	A2	P1	P2
Age (years)	22.3 ± 2.7	23.2 ± 3.3	23.7 ± 3.0	23.1 ± 3.1
BMI (kg/m^2^)	23.0 ± 3.2	21.8 ± 3.1	23.8 ± 3.6	23.0 ± 3.7
Total testosterone (ng/dL)	628.2 ± 208.6	633.9 ± 173.9	618.8 ± 137.4	632.5 ± 197.8
Cortisol (μg/dL)	10.2 ± 4.5	10.7 ± 3.6	10.7 ± 2.6	10.6 ± 2.4
Bench press 1-RM (kg)	51.9 ± 10.0	52.5 ± 9.8	53.2 ± 15.3	53.1 ± 10.9
Leg extension 1-RM (kg)	70.4 ± 12.9	68.9 ± 10.5	71.8 ± 11.9	69.4 ± 14.5

Values are means ± standard deviation (SD) of the randomized population at study initiation (*n* = 30). BMI, body mass index. A1 (SMI-425), A2 (SMI-850), P1 (Placebo-1 set RET), P2 (Placebo-2 sets RET).

**TABLE 2:** Measures of muscle strength and body weight at baseline, 14, 28, and 56 days of treatment, interaction effects, effect sizes, within-group, and between-group comparisons.

Parameter	Group	Evaluation days				Time × group interaction *P*-value Partial Effect size (*η*^2^)
		Baseline ± SD	Day 14 ± SD	Day 28 ± SD	Day 56 ± SD	
1-RM bench press (kg)	A1	52.1 ± 10.1	58.1 ± 10.7^#^	64.0 ± 13.3*^#^	71.5 ± 17.5*^#^	*P* < 0.001^‡^ *η*^2^ = 0.181 (L)
	A2	51.6 ± 8.9	57.0 ± 9.0^#^	64.4 ± 11.6*^#^^	74.2 ± 16.3*^#^^	
	P1	53.2 ± 15.3	55.8 ± 15.5	59.4 ± 15.2*	62.0 ± 15.3*	
	P2	53.5 ± 11.0	57.8 ± 11.6	60.7 ± 9.7*	66.1 ± 11.6*	
1-RM leg extension (kg)	A1	71.0 ± 12.1	80.0 ± 10.2^#^	84.4 ± 9.4*^#^^	87.9 ± 8.5*^#^^	*P* = 0.007^‡^ *η*^2^ = 0.100 (M)
	A2	68.6 ± 10.7	77.2 ± 11.4^#^	83.2 ± 11.2*^#^^	88.0 ± 9.2*^#^^	
	P1	71.8 ± 11.9	74.8 ± 12.9	76.8 ± 13.5*	80.2 ± 13.7*	
	P2	69.1 ± 14.8	75.2 ± 14.2	76.7 ± 12.9*	80.4 ± 11.8*	
Total muscle strength (kg)	A1	123.1 ± 20.3	138.1 ± 17.8^#^	148.5 ± 19.3*^#^^	159.4 ± 22.6*^#^^	*P* < 0.001^‡^ *η*^2^ = 0.217 (L)
	A2	120.2 ± 17.7	134.2 ± 17.8^#^	147.6 ± 20.6*^#^^	162.2 ± 22.9*^#^^	
	P1	125.0 ± 24.0	130.6 ± 24.9	136.2 ± 24.9*	142.2 ± 25.6*	
	P2	122.6 ± 21.4	133.0 ± 22.8	137.4 ± 19.7*	146.5 ± 19.7*	
Body weight (kg)	A1	65.3 ± 11.8	65.5 ± 11.5	65.6 ± 11.3	65.4 ± 11.1	*P* = 0.231 *η*^2^ = 0.042 (S)
	A2	61.7 ± 12.7	61.8 ± 12.4	61.8 ± 12.3	61.9 ± 12.0	
	P1	67.2 ± 13.8	67.1 ± 13.9	67.3 ± 13.8	67.4 ± 13.8	
	P2	63.7 ± 11.6	64.1 ± 12.4	64.2 ± 11.5	64.6 ± 11.3	

Data presented as mean ± standard deviation of measured values at each time point. Significance is considered *p* < 0.05 after mixed factorial repeated measure ANCOVA adjusted by baseline measure as a covariate with Bonferroni correction for multiple comparisons.

^‡^Indicates significant time × group interaction effect and partial effect size (*η*^2^) defined as small, 0.01 (S); moderate, 0.06 (M); and large, 0.14 (L).

*Indicates within-group (time) significance on the difference from adjusted baseline mean (day 14),

^#^indicates a significant difference of group adjusted means compared with P1;

^indicates a significant difference of group adjusted means compared with P2. A1 (SMI-425, *n* = 26), A2 (SMI-850, *n* = 25), P1 (Placebo-1 set RET, *n* = 25), P2 (Placebo-2 sets RET, *n* = 23).

### Muscle strength

Analysis of BP strength as measured by 1-RM is presented in Tables 2, 3. The analysis demonstrated a significant main effect of time (*p* = 0.008, η^2^ = 0.064), group (*p* < 0.001, η^2^ = 0.202), and time × group interaction (*p* < 0.001, η^2^ = 0.181). All groups significantly (*p* < 0.05) improved BP strength from their baseline measures (Table 2), but supplementation improved strength compared to the exercise-only groups. Compared to P1, A1 lifted 3.3 ± 1.1 kg (95% CI 0.5–6.2, *p* = 0.012) more on day 14, 5.7 ± 1.8 kg (95% CI 0.7–10.6, *p* = 0.016) more on day 28, and 10.6 ± 3.0 kg (95% CI 2.6–18.6, *p* = 0.003) more on day 56 (Table 3). A2 increased BP strength compared to both P1 and P2. A2 lifted 6.5 ± 1.9 kg (95% CI 1.5–11.5, *p* = 0.004) more on day 28, and 13.8 ± 3.0 kg (95% CI 5.7–21.8, *p* < 0.001) more on day 56 than P1 (Table 3). A2 bench pressed 5.5 ± 1.9 kg (95% CI 4.1–10.6, *p* = 0.027) more on day 28 and 9.9 ± 3.1 kg (95% CI 1.7–18.2, *p* = 0.010) more on day 56 than P2 (Table 3). The difference in BP strength between A1 and A2 was not significant at any time point. Likewise, P1 and P2 were not statistically different at any time point.

**TABLE 3:** Between-group comparisons of muscle strength mean differences at days 14, 28, and 56 of treatment.

			Evaluation day								
			Day 14			Day 28			Day 56		
**Parameter**	**Group comparisons**		**Mean difference ± SE**	**95% CI**	***P*-value**	**Mean difference ± SE**	**95% CI**	***P*-value**	**Mean difference ± SE**	**95% CI**	***P*-value**
1-RM bench press (kg)	A1	P1	3.3 ± 1.1*	0.5, 6.2	0.012	5.7 ± 1.8*	0.7, 10.6	0.016	10.6 ± 3.0*	2.6, 18.6	0.003
		P2	1.6 ± 1.1	−0.1, 4.5	0.853	4.7 ± 1.9	−0.4, 9.7	0.087	6.8 ± 3.0	−1.4, 15.0	0.166
	A2	P1	2.8 ± 1.1	−0.9, 5.6	0.063	6.5 ± 1.9*	1.5, 11.5	0.004	13.8 ± 3.0*	5.7, 21.8	< 0.001
		P2	1.0 ± 1.1	−1.9, 3.9	1.000	5.5 ± 1.9*	4.1, 10.6	0.027	9.9 ± 3.1*	1.7, 18.2	0.010
1-RM leg extension (kg)	A1	P1	5.9 ± 1.6*	1.5, 10.3	0.003	8.3 ± 1.9*	3.2, 13.3	< 0.001	8.2 ± 2.2*	2.4, 14.0	< 0.001
		P2	3.2 ± 1.7	−1.3, 7.7	0.331	6.3 ± 1.9*	1.1, 11.5	0.009	6.3 ± 2.2*	0.4, 12.2	0.030
	A2	P1	5.1 ± 1.6*	0.7, 9.6	0.014	8.9 ± 1.9*	3.7, 14.0	< 0.001	9.8 ± 2.2*	3.9, 15.7	< 0.001
		P2	2.4 ± 1.7	−2.1, 6.9	0.893	6.9 ± 1.9*	1.6, 12.1	0.004	7.9 ± 2.2*	1.9, 13.9	0.003
Total muscle strength (kg)	A1	P1	9.3 ± 2.1*	3.7, 14.8	< 0.001	14.0 ± 2.9*	6.1, 21.8	< 0.001	18.9 ± 4.0*	8.1, 29.7	< 0.001
		P2	4.6 ± 2.1	−1.1, 10.3	0.191	10.7 ± 3.0*	2.6, 18.7	0.003	12.5 ± 4.1*	1.5, 23.5	0.018
	A2	P1	8.1 ± 2.1*	2.5, 13.7	< 0.001	15.7 ± 3.0*	7.7, 23.6	< 0.001	24.1 ± 4.1*	13.2, 35.1	< 0.001
		P2	3.4 ± 2.1	−2.3, 9.2	0.673	12.3 ± 3.0*	4.2, 20.4	< 0.001	17.7 ± 4.1*	6.6, 28.9	< 0.001

Data presented as mean difference, standard error (SE), and 95% confidence interval (CI) of marginal means after mixed factorial repeated measure ANCOVA, adjusted by baseline measure as a covariate, and Bonferroni correction for multiple comparisons.

*Indicates the mean difference between specified groups is significant at *p* < 0.05. A1 (SMI-425, *n* = 26), A2 (SMI-850, *n* = 25), P1 (Placebo-1 set RET, *n* = 25), P2 (Placebo-2 sets RET, *n* = 23).

Analysis of LE strength demonstrated a significant main effect of time (*p* < 0.001, η^2^ = 0.264), group (*p* < 0.001, η^2^ = 0.245), and time × group interaction (*p* = 0.007, η^2^ = 0.100). Measured values and pairwise group comparisons are shown in Tables 2, 3. All groups significantly (*p* < 0.05) improved over time in LE strength from their baseline values (Table 2), but the supplemented groups lifted more weight than the exercise-only groups. A1’s LE strength measures were greater than that of P1 at each time point, with A1 lifting 5.9 ± 1.6 kg (95% CI 1.5–10.3, *p* = 0.003) more on day 14, 8.3 ± 1.9 kg (95% CI 3.2–13.3, *p* < 0.001) more on day 28, and 8.2 ± 2.2 kg (95% CI 2.4–14.0, *p* < 0.001) more on day 56 (Table 3). A1 also improved more than P2 (*p* < 0.05) on days 28 and 56 (Table 3). A2, the group supplemented with the higher amount of SMI, demonstrated significantly (*p* < 0.05) more strength compared to P1 at all time points and compared to the placebo group assigned to double RET sets, P2, on days 28 and 56 (mean difference of 6.9 ± 1.9 kg, 95% CI 1.6–12.1, *p* = 0.004 and 7.9 ± 2.2 kg, 95% CI 1.9–13.9, *p* = 0.003, respectively; Table 3). Measures of LE strength were not statistically different between A1 and A2, nor between P1 and P2 at any time point.

There was a significant main effect of time (*p* < 0.001, η^2^ = 0.129), group (*p* < 0.001, η^2^ = 0.322), and time × group interaction (*p* < 0.001, η^2^ = 0.217) for total muscle strength. Measured values and pairwise group comparisons are illustrated in Tables 2, 3. Within-group comparisons illustrated that all groups significantly improved over their baseline values in total muscle strength (Table 2). Both supplemented groups, A1 and A2, improved their total muscle strength compared to P1 (*p* < 0.001) at all measured time points (Table 3). A1 improved more than P2 on days 28 (*p* = 0.003) and 56 (*p* = 0.018). A2 also improved (*p* < 0.001, Table 3) total muscle strength compared to P2 on day 28 (mean difference of 12.3 ± 3.0 kg, 95% CI 4.2–20.4) and day 56 (17.7 ± 4.1 kg, 95% CI 6.6–28.9). Total muscle strength between A1 and A2 and between P1 and P2 were not statistically different at any time point.

### Muscle endurance

All groups demonstrated improved muscular endurance as the number of BP-RTF and LE-RTF increased over time (*p* < 0.001 for each group, Table 4). Non-parametric Kruskal–Wallis analyzed at each time point illustrated a significant group effect for BP-RTF on day 28 (*p* = 0.040) and day 56 (*p* = 0.003, Table 4). A2 significantly (*p* < 0.05) improved BP-RTF compared to P1 on days 28 and 56 and compared to P2 on day 56 (Table 4). For LE-RTF, a treatment effect trended toward significance on day 28 (*p* = 0.071) but only reached significance (*p* = 0.005) on day 56. Pairwise comparisons illustrated that both supplemented groups, A1 and A2, improved LE-RTF compared to P1 (*p* < 0.05) but not P2 at day 56 (Table 4).

**TABLE 4:** Within-group and between-group comparisons of bench press and leg extension repetitions at baseline and days 14, 28, and 56 of treatment.

Parameter	Group		Evaluation days				Friedman’s analysis statistic χ^2^ *P*-value
			Baseline	Day 14	Day 28	Day 56	
Bench press repetitions	A1	Mean ± SD	6.7 ± 1.1	9.9 ± 2.6	11.3 ± 2.9	13.4 ± 3.8	*p* < 0.001^‡^
		Median	6.0	9.0*	10.0*	12.0*	
	A2	Mean ± SD	6.6 ± 0.8	9.4 ± 1.6	11.5 ± 2.0	13.9 ± 2.2	
		Median	6.0	9.0	11.0*^#^	14.0*^#^^	
	P1	Mean ± SD	6.6 ± 1.0	8.6 ± 2.2	10.1 ± 2.6	11.3 ± 2.9	
		Median	6.0	8.0	10.0*	12.0*	
	P2	Mean ± SD	6.6 ± 1.0	8.6 ± 1.4	9.9 ± 1.8	11.4 ± 2.0	
		Median	7.0	9.0	10.0*	12.0*	
Kruskal–Wallis Test (H) *P*-value			0.998	0.141	0.040^‡^	0.003^‡^	
Leg extension repetitions	A1	Mean ± SD	7.4 ± 0.6	11.1 ± 2.3	12.6 ± 2.3	15.0 ± 2.0	*p* < 0.001^‡^
		Median	7.5	10.5*	12.0*	15.0*^#^	
	A2	Mean ± SD	6.8 ± 1.0	10.2 ± 2.1	12.8 ± 2.4	15.1 ± 3.2	
		Median	7.0	10.0	13.0*	15.0*^#^	
	P1	Mean ± SD	7.4 ± 1.3	9.7 ± 2.4	11.2 ± 2.6	12.4 ± 2.9	
		Median	7.0	9.0	12.0*	13.0*	
	P2	Mean ± SD	7.2 ± 1.1	10.0 ± 1.7	11.7 ± 1.9	13.3 ± 2.0	
		Median	7.0	10.0*	12.0*	14.0*	
Kruskal–Wallis test (H) *P*-value			0.055	0.102	0.071	0.005^‡^	

Data presented as mean ± standard deviation (SD) and median values. Significance is considered *p* < 0.05 after Friedman repeated measure ANOVA by ranks analysis (for time) and Kruskal–Wallis ANOVA analysis (for group) adjusted by Bonferroni correction for multiple comparisons.

^‡^Indicates significant main effect.

*Indicates within group significance (vs. baseline),

^#^indicates significance compared with P1;

^indicates significance compared with P2; A1 (SMI-425, *n* = 26), A2 (SMI-850, *n* = 25), P1 (Placebo-1 set RET, *n* = 25), P2 (Placebo-2 sets RET, *n* = 23).

### Serum hormones

The baseline and day 56 values of serum hormones are presented in Table 5. Statistical analysis revealed a significant main effect of time (*p* < 0.001, η^2^ = 0.292) and a significant time × group interaction effect (*p* = 0.016, η^2^ = 0.103) for FT values. There was also a significant time (*p* < 0.001, η^2^ = 0.193) and time × group interaction effect evident for serum TT (*p* = 0.017, η^2^ = 0.102). Both supplemented groups (A1, A2) but neither of the exercise-only placebo groups (P1, P2) significantly (*p* < 0.05) increased mean FT and TT values from baseline to day 56 (Table 5). The final FT and TT values for A1 and A2 were significantly (*p* < 0.05) elevated compared to those of P1, but not compared to P2 (Table 5).

**TABLE 5:** Measured serum concentrations of hormones before and after 56 days of treatment, effects, effect sizes, within-group, and between-group comparisons.

		Evaluation days		Time × group interaction *P*-value Partial effect size = *η*^2^
		Baseline	Day 56	
Free testosterone (ng/dL)	A1	2.13 ± 0.88	2.79 ± 0.80*^#^	*P* = 0.016^‡^ *η*^2^ = 0.103 (M)
	A2	1.97 ± 0.79	2.72 ± 0.89*^#^	
	P1	2.09 ± 0.69	2.29 ± 0.41	
	P2	2.07 ± 0.38	2.32 ± 0.58	
Total testosterone (ng/dL)	A1	634.5 ± 222.8	728.3 ± 184.7*^#^	*P* = 0.017^‡^ *η*^2^ = 0.102 (M)
	A2	626.2 ± 178.4	736.8 ± 184.6*^#^	
	P1	617.0 ± 130.2	632.2 ± 126.2	
	P2	640.0 ± 210.3	664.0 ± 166.9	
Cortisol (μg/dL)	A1	10.3 ± 4.5	9.4 ± 3.2	*P* = 0.034^‡^ *η*^2^ = 0.087 (M)
	A2	10.7 ± 3.7	9.3 ± 3.8^	
	P1	10.7 ± 2.6	11.5 ± 3.2	
	P2	10.5 ± 2.4	11.7 ± 3.8	

Data presented as mean ± standard deviation (SD) of measured values at each time point. Significance is considered *p* < 0.05 after repeated measure ANOVA and Bonferroni correction applied for multiple comparisons;

^‡^indicates significant time × group interaction and partial effect size (*η*^2^) defined as small, 0.01 (S); moderate, 0.06 (M); and large, 0.14 (L).

*Indicates within-group (time) significance.

^#^Indicates significance compared with P1 and

^indicates significance compared with P2. A1 (SMI-425, *n* = 26), A2 (SMI-850, *n* = 25), P1 (Placebo-1 set RET, *n* = 25), P2 (Placebo-2 sets RET, *n* = 23).

Analysis of mean C values demonstrated a significant time × group interaction effect (*p* = 0.034, η^2^ = 0.087; Table 5). There was not a significant main effect of time nor were there significant changes from baseline values in any group. However, the analysis revealed a significant (*p* < 0.05) difference between A2 and P2, as A2 decreased from measured baseline values while P2 increased.

## Discussion

This randomized, double-blind, placebo-controlled study investigated the ability of two doses of SMI in conjunction with 8 weeks of RET to augment changes in muscle strength and endurance when compared to non-supplemented, exercising-only, placebo groups. As expected of untrained subjects undertaking a systematic, progressive RET program (23), each randomized group, regardless of assignment, demonstrated a mean improvement in their measured muscular strength in as early as 14 days. However, subjects randomized to receive daily supplementation of SMI gained more muscle strength in comparison to exercising-only placebo groups. Likewise, the mean number of RTF for BP and LE improved in all groups over their baseline values, but those supplemented with SMI further improved these endurance outcomes compared to placebo groups. Overall, these results support the notion that both doses of SMI may help improve muscular adaptation if used alongside overload resistance training.

A curious aspect of RET is that individuals following the same extended, systematic training plan can experience substantially different muscular outcomes. Studies have consistently reported a continuum of muscular adaptation, ranging from negligible to large muscular changes, despite all subjects completing the same training regimen (11, 24, 25). In this study, all subjects completed the same or a more intense training plan, and the overall strength measure improvements ranged from 13.8% (P1) to 34.9% (A2) over their respective baseline values. Within this range, the highest strength gains were found exclusively within the two supplemented groups, suggesting SMI supplementation may increase an individual’s likelihood of responding to a particular RET program with more effective skeletal-muscular adaptation, at least in this subject population of untrained, healthy young men. The precise mechanisms behind the interindividual variability in the adaptive response to RET are not yet clearly elucidated. Nutrition, sex, age, and training variables do not appear to reliably override an individual’s innate muscle response to RET (11, 25–29), suggesting the molecular underpinnings responsible for optimizing muscle responses to RET likely exist downstream from the mechanical loading stimulus, amongst the complex signaling cascades that drive muscle and myofibril protein synthesis (7, 9, 26, 30). Such signals may involve enhancing nitric oxide (NO) release or decreasing reactive oxygen species (ROS) to improve mitochondrial efficiencies and better support the energy-intensive process of post-RET protein synthesis. Few studies have investigated the effects of RET on mitochondrial function and oxidative potential, but tighter coupling of oxidative phosphorylation within skeletal muscle after RET programs in untrained adults has been reported (31, 32). Additionally, studies have shown those individuals showing the highest mitochondrial activities also respond most effectively to RET (33, 34) supporting the notion that improved mitochondrial dynamics and overall improved oxidative capacity may support better adaptive responses to RET.

Mangiferin, a prominent constituent within SMI, is a glucosyl xanthone compound shown in rat models to support mitochondria, assist with enhanced muscle oxidative capacity, and benefit overall skeletal muscle competence (35, 36). Mangiferin may also promote the activation of NO within vascular endothelial cells (37). NO is a well-known signaling molecule that can influence mitochondrial function and modulate skeletal muscle activity (38, 39), while NO precursors have been suggested to stimulate muscle protein synthesis and muscle growth, particularly when combined with exercise (40–42). Additionally, the extracts composing SMI, *Mangifera indica* and *Sphaeranthus indicus*, are both known for their antioxidant properties (18, 19) and may help scavenge oxidants generated in the actively contracting muscle. Excessive ROS can damage mitochondrial DNA, elicit mitoptosis and mitophagy (43), promote overall tissue dysfunction, and accelerate skeletal muscle proteolysis (44). However, compelling counterevidence exists showing antioxidant supplementation negatively impacts RET-induced muscle strength and post-exercise ROS can facilitate skeletal muscle adaptation (45, 46). A limitation to the present study exists as there was no evaluation of ROS or NO in the exercising subjects and further research is required to explore potential mechanisms by which SMI might impact skeletal muscle tissue.

SMI also appears to influence androgenic signaling within muscle tissue as supplemented groups demonstrated significant increases in T. T has anabolic functions within the skeletal muscle, so a transient elevation might exert positive effects on strength (47, 48). T can also elicit an increase in intracellular calcium (49) to temporarily elevate the maximum force production (50) for greater training intensity. While the elevation of TT and FT in the supplemented groups suggests that these hormones could be an important driver of the improved muscular adaptation apparent after supplementation, RET has been shown to have no or limited transient elevation of T in women (51) despite relative changes in strength in women can match or even outperform men (11, 25, 52). This study is limited by the fact that it only included male subjects. As such, further study would be worthwhile to investigate how SMI might affect muscular adaptive responses to RET in women in general, and in peri- and post-menopausal periods characterized by reduced basal T, specifically (53).

The effect of T in exercising muscle is also influenced by the levels of circulating C. C is a catabolic hormone that plays a fundamental role in skeletal muscle adaptive responses to RET by promoting lipolysis and proteolysis (54) and a higher T/C ratio represents an improved balance between the anabolic and catabolic status of the body. A decreasing T/C ratio is frequently used as a marker of exercise-induced stress, physiological strain, and inadequate post-exercise recovery (55). Both placebo groups showed slightly increased C levels in this study, but surprisingly, C decreased in the supplemented groups. The overall elevated T/C ratio in supplemented subjects suggests SMI may improve adaptation to RET regimens. While increased carbohydrate supplementation has been shown to help blunt cortisol response during RET (56), profound between-group dietary differences were unlikely to contribute to these findings considering the subjects were randomized between four groups and three-day food diaries obtained at the study start and end indicated stable dietary patterns. Additionally, there were no significant weight differences between groups at any measured time point. Nonetheless, future research involving SMI should include capturing total caloric intake and detailed dietary macronutrient composition.

Overall, results suggest that daily supplementation with either 425 or 850 mg of SMI was well-tolerated and accentuated muscle adaptation when used by healthy young men participating in a progressive 8-week RET program. The supplemented groups showed significant increases in muscular strength and endurance, increased T, and reduced C in comparison to the two placebo groups, one performing a matching number and the other performing double the number of RET sets. Utilizing a second placebo group lends strength to this study as it illustrates that the significant differences between the active and placebo groups were not simply the results of different work efforts, but rather affirms the notion that cell signaling downstream from the mechanical loading stimulus was altered so that the efficacy of SMI was comparable to performing an additional set of exercises in untrained subjects. These results suggest that SMI might be beneficial to other populations seeking to accentuate muscle responses to RET, for example, those individuals who habitually participate in RET but for whom adaptive responses have stalled (2, 3). Moreover, muscle strength and functionality improve a wide array of health outcomes, so further research should be conducted to investigate if similar outcomes are seen in older adults, where muscular atrophy and sarcopenia are increasingly shown to contribute to reduced functional capacity, increased frailty, and progressive disability (4, 6).

## Data availability statement

The original contributions presented in this study are included in the article/Supplementary material, further inquiries can be directed to the corresponding author.

## Ethics statement

The studies involving humans were approved by the Institutional Ethics Committee (IEC) of the Vydehi Institute of Medical Sciences and Research Center. The studies were conducted in accordance with the local legislation and institutional requirements. The participants provided their written informed consent to participate in this study.

## Author contributions

DS: Visualization, Writing – original draft, Writing – review & editing. SS: Investigation, Supervision, Writing – review & editing. KS: Investigation, Methodology, Supervision, Writing – review & editing. DD: Data curation, Methodology, Project administration, Writing – review & editing. PN: Conceptualization, Methodology, Supervision, Writing – review & editing.
