# Supplementary material for: A botanical extract blend of Mangifera indica and Sphaeranthus indicus combined with resistance exercise training improves muscle strength and endurance over exercise alone in young men: a randomized, blinded, placebo-controlled trial
Source: Front Nutr. 2024 May 3;11:1393917. doi: 10.3389/fnut.2024.1393917 (PMC11099261; doi:10.3389/fnut.2024.1393917)
Supplement: Supplementary file 1 [file Table_1.DOCX]

Table S1: Hematologic parameters

| Parameter | Evaluation  day | A1  (n=26) | A2  (n=26) | P1  (n=25) | P2  (n=24) |
| --- | --- | --- | --- | --- | --- |
|  |  |  |  |  |  |
| WBC  (cells/µL) | Screening | 7.65 ± 1.61 | 7.12 ± 1.83 | 7.02 ± 1.67 | 7.29 ± 1.61 |
|  | Day 56 | 7.44 ± 1.37 | 7.30 ± 1.63 | 7.54 ± 1.74 | 7.32 ± 1.56 |
| RBC (µL) | Screening | 5.43 ± 0.47 | 5.56 ± 0.56 | 5.41 ± 0.36 | 5.61 ± 0.59 |
|  | Day 56 | 5.35± 0.51 | 5.54 ± 0.48 | 5.44 ± 0.32 | 5.35 ± 0.43 |
| Hemoglobin  (g/dL) | Screening | 15.45 ± 1.42 | 15.13 ± 0.99 | 15.71 ± 0.86 | 15.60 ± 0.98 |
|  | Day 56 | 15.48 ± 1.47 | 15.26 ± 0.94 | 15.78 ± 0.94 | 15.38 ± 1.02 |
| Hematocrit  (%) | Screening | 47.36 ± 3.45 | 46.53 ± 2.89 | 47.96 ± 2.30 | 47.83 ± 3.01 |
|  | Day 56 | 46.95 ± 3.41 | 46.93 ± 2.43 | 47.90 ± 2.52 | 46.63 ± 3.03 |
| MCV  (fL) | Screening | 87.65 ± 7.87 | 84.49 ± 9.14 | 88.84 ± 6.42 | 85.95 ± 7.83 |
|  | Day 56 | 87.80 ± 9.12 | 84.70 ± 7.44 | 88.03 ± 6.00 | 86.80 ± 7.54 |
| MCH  (pg/cell) | Screening | 28.63 ± 3.16 | 27.49 ± 3.08 | 29.15 ± 2.45 | 28.06 ± 2.85 |
|  | Day 56 | 28.86 ± 3.49 | 27.77 ± 2.69 | 29.03 ± 2.46 | 28.60 ± 2.67 |
| MCHC  (g/dL) | Screening | 32.60 ± 1.06 | 32.53 ± 0.49 | 32.78 ± 0.69 | 32.63 ± 0.61 |
|  | Day 56 | 32.6 ± 91.07 | 32.63 ± 0.60 | 32.80 ± 0.74 | 32.94 ± 0.56 |
| Platelet  (cells/µL) | Screening | 273.70 ± 56.90 | 270.90 ± 46.50 | 286.80 ± 76.67 | 274.30 ± 64.58 |
|  | Day 56 | 277.50 ± 61.18 | 281.90 ± 50.90 | 300.30± 82.69 | 290.10 ± 45.79 |
| MPV  (μm^3^) | Screening | 8.67 ± 1.02 | 8.53 ± 0.87 | 8.48 ± 0.96 | 8.65 ± 1.43 |
|  | Day 56 | 8.55 ± 0.95 | 8.60 ± 0.74 | 8.28 ± 0.90 | 8.70 ± 1.19 |
| Neutrophils  (%) | Screening | 52.61 ± 9.58 | 50.35 ± 6.25 | 52.80 ± 5.93 | 51.80 ± 8.21 |
|  | Day 56 | 54.78 ± 9.71 | 55.24 ± 10.17 | 55.94 ± 8.01 | 52.80 ± 7.78 |
| Lymphocytes  (%) | Screening | 35.19 ± 7.89 | 35.21 ± 6.68 | 34.80 ± 5.76 | 35.13 ± 8.12 |
|  | Day 56 | 33.25 ± 9.10 | 31.48 ± 9.29 | 32.05 ± 6.67 | 33.89 ± 7.80 |
| Monocytes  (%) | Screening | 6.81 ± 2.24 | 7.90 ± 1.55 | 7.63 ± 1.74 | 7.73 ± 2.11 |
|  | Day 56 | 7.02 ± 1.89 | 7.94 ± 1.94 | 7.34 ± 1.78 | 7.82 ± 1.60 |
| Eosinophils  (%) | Screening | 5.02 ± 3.40 | 6.01 ± 4.38 | 4.36 ± 2.67 | 5.03 ± 3.67 |
|  | Day 56 | 4.40 ± 3.03 | 4.53 ± 4.39 | 3.91 ± 2.56 | 5.54 ± 4.13 |
| Basophils  (%) | Screening | 0.37 ± 0.15 | 0.53 ± 0.39 | 0.41 ± 0.17 | 0.55 ± 0.19 |
|  | Day 56 | 0.38 ± 0.13 | 0.40 ± 0.17 | 0.43 ± 0.12 | 0.55 ± 0.29 |

Data present as mean ± standard deviation of subjects who completed the course of study (n=101). A1 (SMI-425, n=26), A2 (SMI-850, n=26), P1 (Placebo-1, n=25), P2 (Placebo-2, n=24); WBC, white blood cells; RBC, red blood cells; MCV, mean corpuscular volume; MCH, mean corpuscular hemoglobin; MCHC, mean corpuscular hemoglobin concentration; MPV, mean platelet volume.

Table S2: Serum biochemistry parameters

| Parameter | Day | A1  (n=26) | A2  (n=26) | P1  (n=25) | P2  (n=24) |
| --- | --- | --- | --- | --- | --- |
|  |  |  |  |  |  |
| Glucose  (mg/dL) | Screening | 83.81 ± 8.42 | 85.88 ± 11.07 | 89.12 ± 10.21 | 84.33 ± 7.01 |
|  | Day 56 | 85.27 ± 10.00 | 83.19 ± 9.49 | 84.48 ± 10.61 | 83.96 ± 6.10 |
| Sodium  (mEq/L) | Screening | 138.5 ± 1.45 | 138.5 ± 2.01 | 138.6 ± 1.71 | 138.5 ± 1.89 |
|  | Day 56 | 137.8 ± 2.21 | 138.0 ± 2.54 | 137.5 ± 2.31 | 137.5 ± 3.01 |
| Potassium (mmol/L) | Screening | 4.15 ± 0.33 | 4.22 ± 0.36 | 4.30 ± 0.45 | 4.24 ± 0.29 |
|  | Day 56 | 4.34 ± 0.46 | 4.39 ± 0.39 | 4.40 ± 0.46 | 4.31 ± 0.35 |
| Urea Nitrogen (mg/dL) | Screening | 10.50 ± 2.89 | 10.31 ± 2.11 | 10.76 ± 3.83 | 10.38 ± 2.83 |
|  | Day 56 | 10.12 ± 3.30 | 10.31 ± 2.68 | 9.32 ± 2.50 | 10.44 ± 2.73 |
| Creatine  (μmol/L) | Screening | 76.04 ± 10.61 | 71.62 ± 12.38 | 72.50 ± 9.73 | 73.39±8.84 |
|  | Day 56 | 76.04 ± 12.38 | 76.04 ± 18.57 | 73.39 ± 15.03 | 69.85 ± 8.84 |
| T. Protein (g/dL) | Screening | 7.44 ± 0.26 | 7.56 ± 0.38 | 7.52 ± 0.31 | 7.46 ± 0.32 |
|  | Day 56 | 7.52 ± 0.42 | 7.55 ± 0.31 | 7.58 ± 0.31 | 7.54 ± 0.29 |
| Albumin  (g/dL) | Screening | 4.42 ± 0.20 | 4.46 ± 0.27 | 4.47 ± 0.26 | 4.40 ± 0.20 |
|  | Day 56 | 4.40 ± 0.27 | 4.44 ± 0.30 | 4.40 ± 0.21 | 4.43 ± 0.23 |
| Total Bilirubin (mg/dL) | Screening | 1.13 ± 0.48 | 0.88 ± 0.30 | 1.08 ± 0.38 | 1.13 ± 0.43 |
|  | Day 56 | 1.03 ± 0.46 | 0.83 ± 0.24 | 1.00 ± 0.32 | 1.09 ± 0.35 |
| Creatine kinase (U/L) | Screening | 141.5 ± 40.00 | 140.5 ± 76.04 | 138.3 ± 48.23 | 123.5 ± 67.18 |
|  | Day 56 | 138.5 ± 43.54 | 146.5 ± 73.54 | 124.6 ± 36.94 | 134.1 ± 85.90 |
| ALP (U/L) | Screening | 77.73 ± 19.19 | 71.58 ± 19.31 | 75.92 ± 38.58 | 71.92 ± 17.38 |
|  | Day 56 | 75.69 ± 16.34 | 76.81 ± 20.89 | 79.84 ± 33.98 | 75.08 ± 15.66 |
| AST (U/L) | Screening | 25.35 ± 8.00 | 22.50 ± 4.09 | 23.76 ± 4.74 | 23.96 ± 4.17 |
|  | Day 56 | 25.96 ± 6.97 | 22.69 ± 3.59 | 22.32 ± 4.53 | 26.42 ± 6.51 |
| ALT (U/L) | Screening | 27.27 ± 18.13 | 19.31 ± 5.89 | 25.40 ± 13.07 | 22.42 ± 7.96 |
|  | Day 56 | 26.46 ± 10.72 | 18.50 ± 3.89 | 23.68 ± 11.23 | 30.54 ± 19.16 |
| T. cholesterol  (mg/dL) | Screening | 151.9 ± 28.28 | 156.2 ± 26.91 | 160.3 ± 37.61 | 160.9 ± 25.90 |
|  | Day 56 | 148.5 ± 26.72 | 152.9 ± 30.52 | 155.0 ± 29.44 | 167.2 ± 22.89 |
| Triglycerides (mg/dL) | Screening | 99.58 ± 56.47 | 106.7 ± 59.71 | 102.6 ± 53.30 | 105.0 ± 63.71 |
|  | Day 56 | 100.8 ± 55.36 | 106.2 ± 50.16 | 104.4 ± 49.67 | 116.9 ± 51.91 |
| HDL  (mg/dL) | Screening | 39.38 ± 10.10 | 41.63 ± 11.14 | 41.18 ± 8.60 | 42.48 ± 12.11 |
|  | Day 56 | 40.11 ± 9.68 | 39.75 ± 9.57 | 39.02 ± 7.81 | 39.74 ± 8.54 |
| LDL  (mg/dL) | Screening | 100.2 ± 25.19 | 101.6 ± 29.21 | 106.2 ± 32.14 | 105.8 ± 16.74 |
|  | Day 56 | 102.5 ±28.63 | 101.1 ± 29.24 | 105.3 ± 28.58 | 114.2 ± 18.37 |

Data present as mean ± standard deviation of subjects who completed the course of the study (n=101). A1 (SMI-425, n=26), A2 (SMI-850, n=26), P1 (Placebo-1, n=25), P2 (Placebo-2, n=24); T, Total; ALP, alkaline phosphatase; ALT, alanine transaminase; AST, aspartate aminotransferase; HDL, high-density lipoprotein; LDL, low-density lipoprotein.
